# Role of microRNAs in the Regulation of α-Synuclein Expression: A Systematic Review

**DOI:** 10.3389/fnmol.2016.00128

**Published:** 2016-11-21

**Authors:** Ariadna Recasens, Celine Perier, Carolyn M. Sue

**Affiliations:** ^1^Department of Neurogenetics, Kolling Institute, The Royal North Shore Hospital, Northern Sydney Local Health DistrictSt. Leonards, NSW, Australia; ^2^Northern Clinical School, Sydney Medical School, University of SydneySydney, NSW, Australia; ^3^Neurodegenerative Disease Laboratory, Vall d’Hebron Research Institute and Centre for Networked Biomedical Research on Neurodegenerative Diseases (CIBERNED)Barcelona, Spain

**Keywords:** Parkinson’s disease, α-synuclein, microRNA, alpha-synuclein, gene expression, miRNA, gene regulation

## Abstract

Growing evidence suggests that increased levels of α-synuclein might contribute to the pathogenesis of Parkinson’s disease (PD) and therefore, it is crucial to understand the mechanisms underlying α-synuclein expression. Recently, microRNAs (miRNAs) have emerged as key regulators of gene expression involved in several diseases such as PD and other neurodegenerative disorders. A systematic literature search was performed here to identify microRNAs that directly or indirectly impact in α-synuclein expression/accumulation and describe its mechanism of action. A total of 27 studies were incorporated in the review article showing evidences that six microRNAs directly bind and regulate α-synuclein expression while several miRNAs impact on α-synuclein expression indirectly by targeting other genes. In turn, α-synuclein overexpression also impacts miRNAs expression, indicating the complex network between miRNAs and α-synuclein. From the current knowledge on the central role of α-synuclein in PD pathogenesis/progression, miRNAs are likely to play a crucial role at different stages of PD and might potentially be considered as new PD therapeutic approaches.

## Introduction

Growing evidence suggests that increased levels of α-synuclein are toxic and may contribute to the pathogenesis of Parkinson’s disease (PD). Supporting evidence includes: (i) duplications and triplication of the α-synuclein gene cause dominantly inherited PD, with a dose-correlation of α-synuclein load to the PD phenotype (Singleton et al., 2003; Ibáñez et al., 2004, 2009; Ahn et al., 2008; Ross et al., 2008); (ii) polymorphisms in α-synuclein promoters are associated with increased PD risk by enhancing α-synuclein expression (Chiba-Falek and Nussbaum, 2001; Touchman et al., 2001; Maraganore et al., 2006); (iii) increased α-synuclein mRNA levels are found in surviving dopaminergic (DA) neurons in the substantia nigra (SN) of idiopathic PD patients (Gründemann et al., 2008); (iv) induced pluripotent stem cells (iPS) from PD patients exhibited α-synuclein accumulation (Nguyen et al., 2011; Sánchez-Danés et al., 2012; Mazzulli et al., 2016); (v) α-synuclein is up-regulated in several *in vivo* PD models including 1-methyl-4-phenyl-1,2,3,6-tetrahydropyridine (MPTP) mice and monkeys (Vila et al., 2000, 2001; Purisai et al., 2005); and (vi) overexpression of human wild-type and A53T mutant α-synuclein in rats and monkeys induced nigrostriatal degeneration (Kirik et al., 2002, 2003). In addition to PD, α-synuclein plays a key role in and other synucleinopathies such as dementia with Lewy bodies (DLB) and multiple system atrophy (MSA; Tagliafierro and Chiba-Falek, 2016).

**Figure 1:**
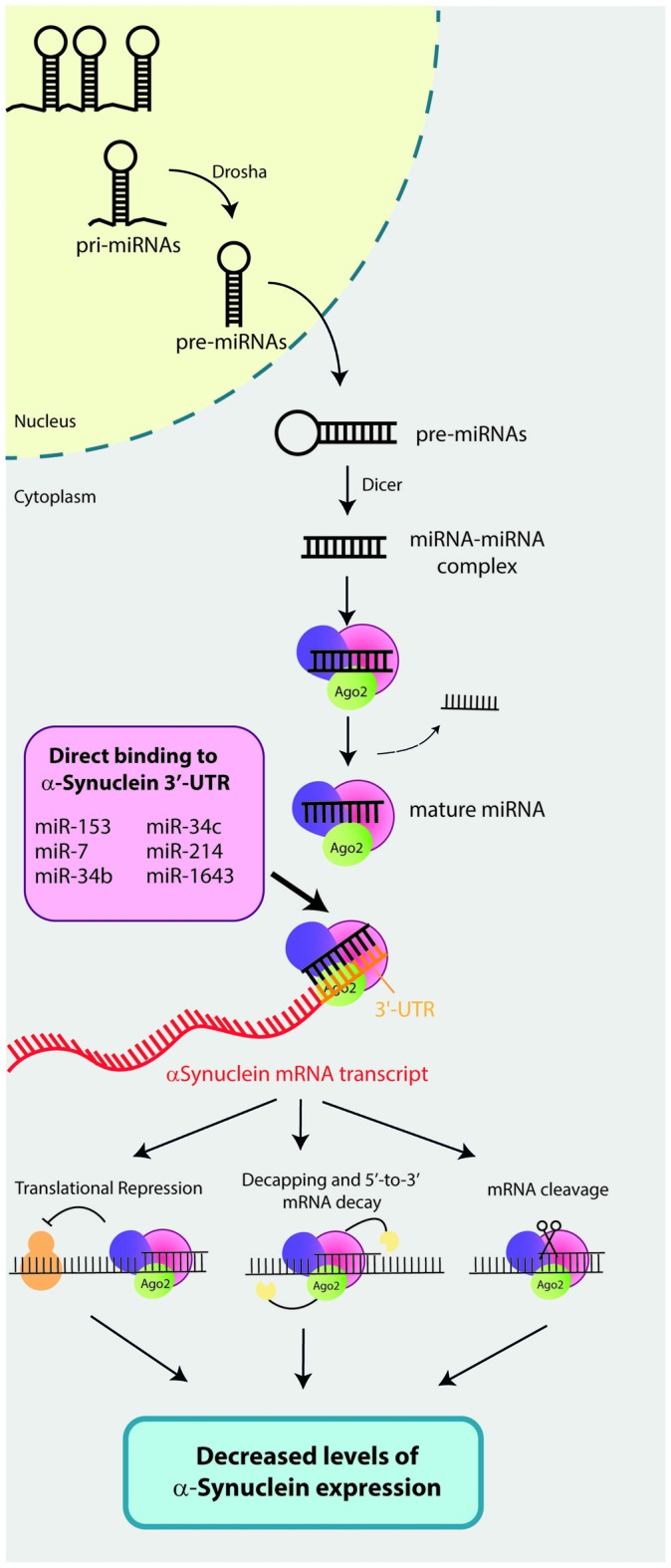
**Mechanisms of action of microRNAs (miRNAs) that directly bind and regulate α-synuclein expression.** Biogenesis of miRNAs required a multi-step process that takes place in the nucleus and the cytoplasm of the cells. First, miRNAs are transcribed in the nucleus by the RNA polymerase II as long primary miRNAs (pri-miRNAs). Then, a RNAse III enzyme called Drosha converts pri-miRNAs into 60 base-pair (bp) stem-loop structures (pre-miRNAs). Pre-miRNAs are subsequently exported to the cytoplasm by the Exportin 5, where a second RNase II enzyme called Dicer will process pre-miRNAs into a ~22nt miRNA-miRNA complex. The RNA-duplex binds to an Argonaute (AGO) protein and one of the strands is removed resulting in the mature RNA-induced silencing complex (RISC). Finally, RISC binds to complementary mRNA sequence (seed matches) and represses its expression by: (i) translational repression; (ii) mRNA decapping and decay; or (iii) direct cleavage of mRNAs target by RISC. To date, six miRNAs have been described to directly bind the 3′-untranslational region (UTR) of the α-synuclein mRNA transcript and repress its expression. These six miRNAs are: miR-7, miR-153, miR-34b, miR-34c, miR-214 and miR-1643.

MicroRNAs (miRNAs) are endogenous 17–24 base-pair (bp) single-stranded non-coding RNAs that have recently emerged as a key regulators of gene expression. Biogenesis of miRNAs, which are encoded within the genome as independent genomic transcription units or as introns of protein-coding genes, required a multi-step process that takes place in the nucleus and the cytoplasm (Figure 1; Meister and Tuschl, 2004; Ameres and Zamore, 2013; Catalanotto et al., 2016). First, miRNAs are transcribed in the nucleus by the RNA polymerase II as long primary miRNAs (pri-miRNAs) which are converted by the RNAse III enzyme Drosha into 60 bp stem-loop structures called pre-miRNAs. The pre-miRNAs are subsequently exported to the cytoplasm by the Exportin 5 via a nuclear pore. Once in the cytoplasm, the pre-miRNAs are further processed by a second RNase II enzyme called Dicer into a ~22nt miRNA-miRNA complex intermediate. Then, the RNA-duplex binds to an Argonaute (AGO) protein and one of the strands is removed resulting in the mature RNA-induced silencing complex (RISC). Finally, the RISC will bind to complementary mRNA sequence (seed matches) and repress their expression by: (i) translational repression via blocking translational initiation, poly(A) tail shortening or recruiting translation blockers; (ii) mRNA decay; or (iii) direct cleavage of target mRNAs by RISC (Ameres and Zamore, 2013). Via regulation of target genes, miRNAs are involved in several biological process including cell proliferation, differentiation, apoptosis, development, angiogenesis and immune response (Huang et al., 2011; Tüfekci et al., 2014a) and therefore, miRNAs dysregulation is associated with the pathogenesis of several human disease such as cancer, diabetes, autoimmune diseases, neurological disorders, diabetes and cardiovascular disease (Tüfekci et al., 2014b).

Considering the importance of modulating α-synuclein levels in PD and other related disorders, the objective of this study is to review miRNAs that impact directly or indirectly in α-synuclein expression and describe their mechanisms of action.

## Materials and Methods

### Registration

Following PRISMA recommendations (Liberati et al., 2009; PRISMA Checklist available in Supplementary Materials), the systematic review was registered in The Joanna Bridge Institute (JBI) website with date 27th July 2016. Registration details are described in Supplementary Table 1.

### Eligibility Criteria

All the studies selected for the review satisfied the PICOS selection criteria detailed in Supplementary Methods. No language or publication date restrictions were imposed.

### Information Sources and Search

Three different database were used in this review article: PubMed, Scopus and Web of Science. The last search was run on 25th May 2016. No supplementary approaches were used to identify additional studies. Duplicated records were removed. The full electronic search strategy for each database is described in Table 1.

**Table 1:** **Full electronic search strategy for each database used in the review article**.

Database	Query	No. records
Pubmed	(“MicroRNAs”[Mesh] OR miRNA OR miRNAs OR microRNA OR MIR) AND (“alpha-Synuclein”[Mesh] OR α-synuclein OR α-synucleins OR alpha-synuclein OR snca OR alphasynuclein OR alphasynucleins OR alpha-synuclein OR alpha-synucleins OR “alpha synuclein” OR “alpha synucleins”)	69
Scopus	(MicroRNAs OR miRNA OR miRNAs OR microRNA OR MIR) AND (alpha-Synuclein OR α-synuclein OR α-synucleins OR alpha-synuclein OR snca OR alphasynuclein OR alphasynucleins OR alpha-synucleins OR “alpha synuclein” OR “alpha synucleins”)	121
Web of science	((MicroRNAs OR miRNA OR miRNAs OR microRNA OR MIR) AND (alpha-Synuclein OR α-synuclein OR α-synucleins OR alpha-synuclein OR snca OR alphasynuclein OR alphasynucleins OR alpha-synucleins OR “alpha synuclein” OR “alpha synucleins”))	163

### Study Selection

First, an over-inclusive screening by titles and abstracts was done to identify potential relevant studies. At this stage, irrelevant records, reviews, abstracts, editorials, letters, comments, perspective, reports, opinion and book chapter were removed. Full-text articles from the candidate studies were read and a second screening was done accordingly to the exclusion criteria detailed in Supplementary Methods.

### Data Extraction

All the included studies were divided into two groups: (i) overexpressing studies: studies using α-synuclein overexpressing models (OEM); and (ii) standard studies. Relevant information from all included studies was extracted using two different extraction datasheets, depending on the category of the article (overexpressing vs. standard). See Supplementary Methods for detailed data extraction.

## Results

**Figure 2:**
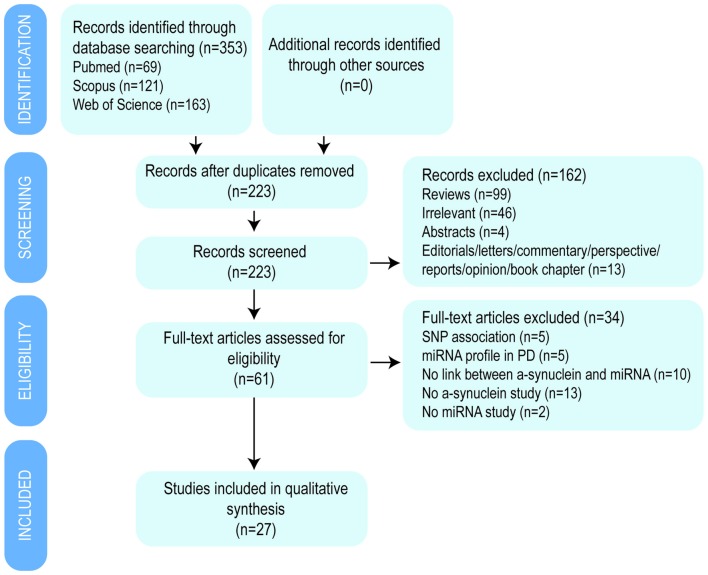
**Flow diagram of the selection process by which the studies were included in the review article**.

Initially, a total of 353 publications were identified using three databases: Pubmed, Scopus and Web of Science (Figure 2). After duplicates removal, a total of 223 studies were screened by title and abstract and 61 potential relevant studies were selected for full-text review. A second screening was performed and 34 studies were discarded according to the following exclusion criteria: five were single nucleotide polymorphism (SNP) association studies with PD, another five were miRNA expression profile studies in PD patients, 12 studies did not investigate the impact on α-synuclein expression, two studies did not describe the impact of miRNAs expression, and 10 studies did not correlate miRNA and α-synuclein expression. Finally, 27 studies were included in the present review article, from which 12 investigations studied the effect of miRNA that directly bind and regulates α-synuclein expression, 10 studies focused on miRNAs that indirectly impact on α-synuclein expression and five studies used α-synuclein overexpressing *in vivo* models (Table 2).

**Table 2:** **Summary of studies included in the review article**.

References	Category	miRNA studied/overexpressing model used
Junn et al. (2009)	Direct	miR-7
Doxakis (2010)	Direct	miR-7 and miR-153
Latreille et al. (2014)	Direct	miR-7
Choi et al. (2014)	Direct	miR-7
Fragkouli and Doxakis (2014)	Direct	miR-7 and miR-153
Fan et al. (2015)	Direct	miR-7
Zhou et al. (2016)	Direct	miR-7
Song et al. (2012)	Direct	miR-7
Kim et al. (2013)	Direct	miR-153
Lim and Song (2014)	Direct	miR-153 and miR-1643
Kabaria et al. (2015)	Direct	miR-34b and miR-34c
Wang et al. (2015)	Direct	miR-214
Alvarez-Erviti et al. (2013)	Indirect	miR-21*; miR-224; miR-373*; miR-379, miR-26b: miR-106a* and miR-301b
Li et al. (2014)	Indirect	miR-320
Su et al. (2016)	Indirect	miR-21
Zhang and Cheng (2014)	Indirect	miR-16-1
Decressac et al. (2013)	Indirect	miR-128
Niu et al. (2016)	Indirect	miR-133
Wang et al. (2008)	Indirect	miR-433
Schmitt et al. (2012)	Indirect	miR-433
Parsi et al. (2015)	Indirect	miR-16
Gillardon et al. (2008)	OEM	Mice model
Asikainen et al. (2010)	OEM	*Caenorhabditis elegans*
Ubhi et al. (2014)	OEM	Mice model
Kong et al. (2015)	OEM	*Drosophila*
Schafferer et al. (2016)	OEM	Mice model
Thome et al. (2016)	OEM	Mice model

*The included studies were divided into three categories: (1) direct: studies associated with miRNAs that directly binds and modulate α-synuclein expression; (2) indirect: studies related with miRNAs that indirectly impact on α-synuclein expression; and (3) overexpressing models (OEM) of α-synuclein*.

### miRNAs that Directly Bind 3′-UTR α-Synuclein and Negatively Regulate α-Synuclein Expression

The results derived from all included studies demonstrated that a total of six miRNAs (miR-7, miR-153, miR-34b, miR-34c, miR-214 and miR-1643) directly bind to the 3′-untranslated region (UTR) of the α-synuclein mRNA transcript and negatively regulate its expression (Figure 1).

#### miR-7

Seven studies demonstrated the impact of miR-7 in α-synuclein expression (Junn et al., 2009; Doxakis, 2010; Choi et al., 2014; Fragkouli and Doxakis, 2014; Latreille et al., 2014; Fan et al., 2015; Zhou et al., 2016). From these studies, a total of three confirmed the direct binding of miR-7 to α-synuclein 3′-UTR sequence using luciferase reporter assays in three different *in vitro* models (SH-SY5Y, HEK293T and primary neurons; Supplementary Table 2). The specificity of the binding site was confirmed by introducing mutations in the α-synuclein 3′-UTR sequence that blocked the effect of miR-7 in the luciferase activity assay. The predicted binding site of miR-7 within the α-synuclein gene required to repress its expression is located at bases 119–217 of the α-synuclein 3′-UTR (Junn et al., 2009; Doxakis, 2010).

The direct effect of miR-7 in α-synuclein expression was first reported by Junn et al. (2009). In particular, transfection with 40 nM of premiR-7-2 in HEK293T cells resulted in a reduction of α-synuclein expression both at protein and mRNA levels. On the other hand, treatment with miR-7 inhibitors significantly increased the levels of α-synuclein protein in SH-SY5Y cells. The direct impact of miR-7 in α-synuclein expression was reproduced by Doxakis (2010) using both HEK293T cells and murine primary neurons.

One of the studies was focused on the role of miR-7 in pancreatic β-cell function (Latreille et al., 2014) and generated a miR-7 conditional knockout mice using Cre/Lox system (miR7a2^fl/fl^ mice) which developed diabetes due to impaired insulin secretion and *β* cell differentiation. The direct impact of miR-7 in α-synuclein expression was confirmed in MIN6 cells and pancreatic islets obtained from miR7a2^fl/fl^ mice. In particular, adenovirus-miR7a-mediated overexpression in MIN6 cells resulted in a reduction of α-synuclein transcript levels, while exposure to miR-7a inhibitors increased α-synuclein mRNA and protein levels. In addition, α-synuclein levels were increased in miR7a2^fl/fl^ pancreatic islets. Interestingly, miR-7 played a role in insulin secretion by repressing the expression of α-synuclein which in turn modulated the granule fusion with the plasma membrane. These results are in line with the previous observation that α-synuclein, whose exact function still remains unknown, plays a role in neurotransmitter release via regulating the pool of vesicles available in the synaptic bouton and its fusion with the plasma membrane (Murphy et al., 2000; Cabin et al., 2002; Fernández-Chacón et al., 2004; Chandra et al., 2005; Larsen et al., 2006; Mazzulli et al., 2016).

The neuroprotective effect of miR-7 has been assessed under different conditions (Junn et al., 2009; Choi et al., 2014; Fragkouli and Doxakis, 2014; Fan et al., 2015). One of the studies investigated the protective effect of miR-7 in N20Y cells overexpressing mutant A53T α-synuclein challenged with hydrogen peroxide (H_2_O_2_). Notably, the presence of miR-7 reduced H_2_O_2_-induced cell death in A53T α-synuclein mutant cells (Junn et al., 2009). Additional protective effects of miR-7 against the MPTP-active metabolite 1-methyl-4-phenylpyridinium (MPP^+^) *in vitro* was investigated in two studies (Choi et al., 2014; Fragkouli and Doxakis, 2014). Both of them demonstrated that overexpression of miR-7 significantly increased cell viability after MPP^+^ treatment in SH-SY5Y cells, ReNcell VM cells and mouse primary neurons. One of the studies suggested that the protective effect of miR-7 against MPP^+^ is independent of α-synuclein repression, since knocking down α-synuclein in SH-SY5Y cells did not impact on miR-7-enhanced cell viability. This study rather suggested that miR-7 protected against MPP^+^-induced cell death by directly targeting the expression of RelA, a component of the nuclear factor kappa-light-chain-enhancer of activated B cells (NF-κB) consequently relieving NF-κB suppression (Choi et al., 2014). On the other hand, Fragkouli and Doxakis (2014) suggested that miR-7 protects against MPP^+^-induced cell death by activating the mTOR pathway. Relevant to this context, SH-SY5Y cells treated with MPP^+^ and subchronic MPTP administration in mice resulted in a significant reduction of miR-7 expression in both models (Junn et al., 2009; Choi et al., 2014; Fragkouli and Doxakis, 2014). Two studies were focused on the protective effect of miR-7 against A53T mutant α-synuclein-induced toxicity (Fan et al., 2015; Zhou et al., 2016). Both studies concluded that miR-7 protects against PD-like degeneration by directly targeting nod-like receptor protein 3 (Nlrp3*)* expression and therefore modulating NLRP3 inflammasome activation. The protective effect of miR-7 *in vivo* was also assessed in the MPTP mice model (Zhou et al., 2016), in which the injection of miR-7 mimics into wild type mice treated with subacute MPTP dose rescued the loss of tyrosine hydroxylase (TH)-positive neuron number in the SN and dramatically inhibited Ionized calcium binding adaptor molecule 1 (Iba1) microglial activation via supressing NLRP3 inflammasome-mediated neuroinflammation. Further supporting the concept that miR-7 regulates α-synuclein expression *in vivo*, Song et al. (2012) reported that schizophrenia-like transgenic mice overexpressing heme oxygenas-1 (HO-1) protein in astrocytes exhibited decreased levels of miR-7 and increased α-synuclein levels in the SN/ventral tegmental area (VTA) at 48-weeks of age compare to control animals.

#### miR-153

A total of four studies investigated the impact of miR-153 in α-synuclein expression and its protective effect (Doxakis, 2010; Kim et al., 2013; Fragkouli and Doxakis, 2014; Lim and Song, 2014). Two studies investigated the combined effects of both miR-7, miR-153 and the combination of miR-7/153 (Doxakis, 2010; Fragkouli and Doxakis, 2014). One study predicted the binding site of miR-153 within the α-synuclein gene in the 442–448 bases of the α-synuclein 3′-UTR (Doxakis, 2010). The specificity of the predicted binding site for miR-153 was confirmed *in vitro* (Doxakis, 2010; Kim et al., 2013; Lim and Song, 2014) using luciferase assays and introducing mutations in the α-synuclein 3′-UTR. The direct effect of miR-153 in α-synuclein expression has now been studied in HEK293T cells. Cotransfection with an α-synuclein plasmid containing the 3′-UTR and miR-153 significantly reduced α-synuclein levels both at protein and mRNA level (Doxakis, 2010). The protective effect of miR-153 was also studied in embryonic murine neurons treated with MPP^+^. As reported with miR-7, overexpression of miR-153 in primary cortical neurons attenuated MPP^+^-induced neurotoxicity by upregulating the mTOR pathway (Fragkouli and Doxakis, 2014).

#### miR-34b and miR-34c

One study demonstrated that miR-34b and miR-34c directly targeted α-synuclein expression (Kabaria et al., 2015). Computational algorithms were used to predict two miR-34b and one miR-34c binding sites in the 3′-UTR of α-synuclein mRNA (Kabaria et al., 2015): miR-34b binding site #1: located between bases 528–549; miR-34b binding site #2: between bases 732–754; and miR-34c binding site: between bases 1149–1171. These bindings sites were verified by cotransfecting SH-SY5Y cells with a plasmid construct expressing α-synuclein 3′-UTR with miR-34b or miR-34c. Interestingly, the introduction of a polymorphic variation (rs10024743) which lies within the target site 1 of miR-34b significantly decreased the impact of miR-34b in the luciferase activity. As a consequence of the direct binding between miR-34b/miR-34c and α-synuclein, overexpression of miR-34b or miR-34c in SH-SY5Y cells resulted in significant reduction in α-synuclein mRNA and protein levels. Interestingly, miR-34b and miR-34c did not repress β-synuclein, but rather increased its expression by up to 2.3-fold. Moreover, inhibition of miR-34b and miR-34c increased α-synuclein mRNA and protein level as well as the formation of α-synuclein-containing aggregates in DA neurons.

#### miR-214

Only one study has demonstrated the direct impact of miR-214 in α-synuclein expression. Using luciferase assays in SH-SY5Y cells, miR-214 has been shown to directly target the α-synuclein 3′-UTR. In addition, miR-214 overexpression reduced α-synuclein expression both at mRNA and protein levels, while downregulation of miR-214 increased not only α-synuclein expression (mRNA and protein) but also the number of α-synuclein-aggregates in cells (Wang et al., 2015). This work also investigated whether the regulation of α-synuclein by miR-214 was the mechanism underlying the neuroprotective effect of Resveratrol. First, they showed that Resveratrol could ameliorate MPP^+^/MPTP-induced cell death both *in vitro* and *in vivo*. Interestingly, miR-214 inhibitors reversed the neuroprotective effect of resveratrol treatment in MPP^+^/MPTP models.

#### miR-1643

One study found that miR-1643 is a direct regulator of α-synuclein expression (Lim and Song, 2014). Luciferase assay in 293TF cells confirmed the direct binding of miR-1643 to α-synuclein 3′-UTR sequence.

### miRNAs that Indirectly Impact on α-Synuclein Expression Without Binding to α-Synuclein 3′-UTR Sequence

**Figure 3:**
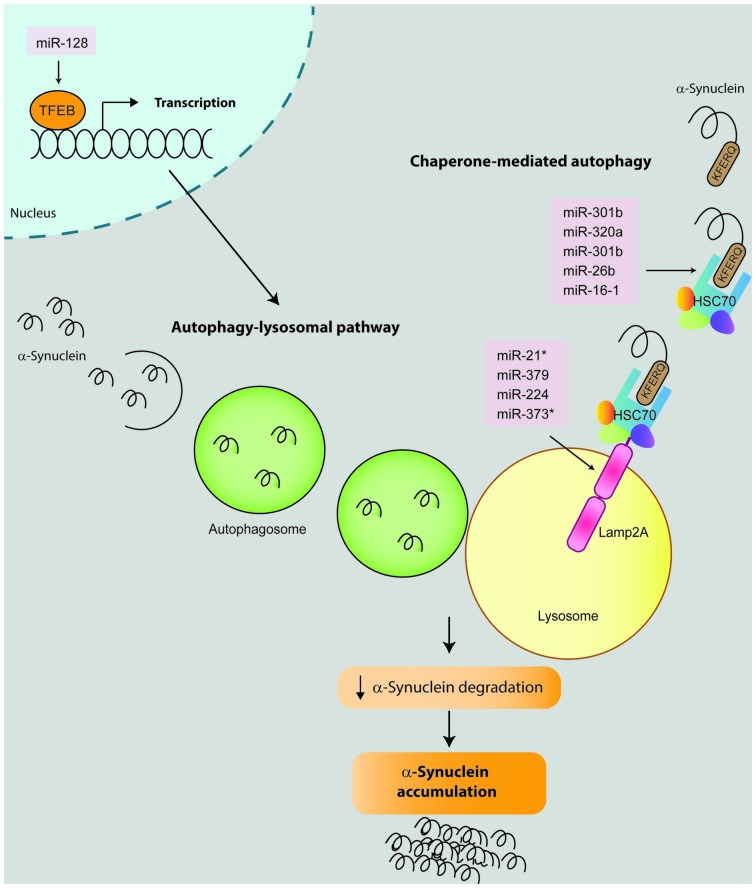
**miRNAs that impact on α-synuclein expression by modulating proteolytic degradation pathways.** α-Synuclein can be degraded by several proteolytic pathways including chaperone-mediated autophagy (CMA) and autophagy-lysosomal pathway (ALP). During the CMA, the KFREQ-like sequence of the α-synuclein protein is recognized by a chaperone complex which includes the *Heat shock protein 70* (Hsc70). This complex is guided to the lysosomes and recognized by the *Lysosome-associated membrane protein 2* (Lamp2A), which in turn translocate the α-synuclein into the lysosome where it is finally degraded by hydrolytic enzymes. To date, nine microRNAs have been described to modulate the CMA pathway and impact on α-synuclein degradation by directly binding and repressing the expression of Hsc70 (miR-301b, miR-26b, miR-320a, miR-106a and miR-16-1) or Lamp2a (miR-21*, miR-379, miR-373* and miR-224). For ALP degradation, α-synuclein is firstly enclosed into an autophagosome. Then the autophagosome is guided and fused with a lysosome where α-synuclein is finally degraded. In this context, miRNA-128 activates transcription factor EB (TFEB) which has been demonstrated to promote the transcription of genes involved in ALP pathway. Therefore miRNA-repression of Hsc7, Lamp2a or TFEB result in alterations in the α-synuclein degradation and its consequent accumulation.

**Figure 4:**
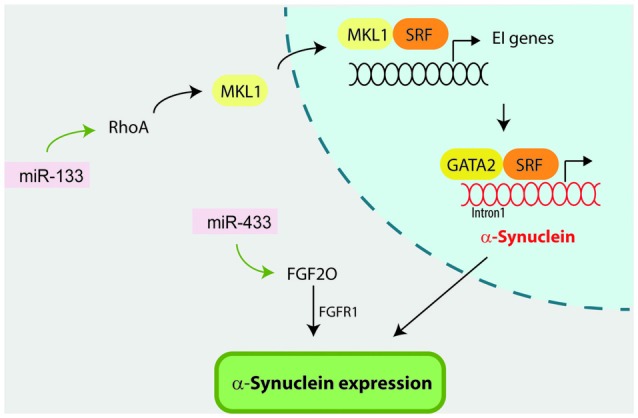
**miR-133 and miR-433 indirectly influence α-synuclein by targeting *Ras homolog gene family, member A* (RhoA) and *fibroblast growth factor 20* (FGF20) respectively.** On one hand, miR-133 targets and regulates RhoA expression which has been previously reported to regulate α-synuclein expression. In this context, RhoA first activates *megakaryoblastic leukemia 1* (MKL-1) factor, which in turn activates *serum response element* (SRF) transcription factor. MKL-1 and SRF activation promote the transcription of early immediate (EI) genes. Finally SRF forms a multiprotein complex with GATA-2 factor which regulates α-synuclein expression via occupancy at the intron-1. On the other hand, miR-433 directly targets FGF20, which has been suggested to directly regulate α-synuclein expression through the FGF-receptor 1 (FGFR1).

In addition to miRNAs that directly bind and regulate α-synuclein protein, several miRNAs have been reported to indirectly regulate α-synuclein levels by targeting the expression of other genes. There are five different studies that report miRNAs that directly impact on proteolytic pathways and result in α-synuclein accumulation (Figure 3; Alvarez-Erviti et al., 2013; Decressac et al., 2013; Li et al., 2014; Zhang and Cheng, 2014; Su et al., 2016). In addition, miR-133b (Niu et al., 2016) and miR-433 (Wang et al., 2008; Schmitt et al., 2012) have been reported to impact on α-synuclein by directly targeting Ras homolog gene family, member A (RhoA) and fibroblast growth factor 20 (FGF20), respectively (Figure 4).

#### miRNAs, Proteolytic System and α-Synuclein

α-Synuclein turnover predominantly involves chaperone-mediated autophagy (CMA). Therefore, alterations in CMA result in pathological α-synuclein accumulation. Four studies have investigated how miRNA regulation of CMA influences α-synuclein accumulation (Alvarez-Erviti et al., 2013; Li et al., 2014; Zhang and Cheng, 2014; Su et al., 2016). Firstly, Alvarez-Erviti et al. (2013) transfected SH-SY5Y cells overexpressing α-synuclein with seven miRNAs that directly bind and negatively regulate two key proteins involved in CMA: Lysosome-associated membrane protein 2 (Lamp2a, hsa-miR-21*; hsa-miR-224; hsa-miR-373*; and hsa-miR-379) and Heat shock protein 70 (Hsc70, hsa-miR-26b: hsa-miR-106a*; and hsa-miR-301b). In addition to the expected reduction in Lamp2a and Hsc70 gene expression, transfection with the seven candidate miRNAs significantly increased α-synuclein protein levels. Notably, only two of them (miR-106a* and miR-301b) caused a significant decrease in α-synuclein mRNA levels. Interestingly, miR-106a* was predicted to target the 3′-UTR of α-synuclein although the direct binding has not yet been confirmed.

The impact of miR-21 on Lamp2a and α-synuclein aggregation was confirmed by a second study using SH-SY5Y cells. Cells transfected with miR-21 mimics exhibited decreased levels of Lamp2a both at protein and mRNA levels, and increased α-synuclein only at the protein level. On the other hand, SH-SY5Y cells treated with miR-21 inhibitors displayed increased levels of Lamp2a (protein and mRNA) and decreased α-synuclein levels. This study also suggested that geniposide had a neuroprotective effect against MPP^+^/MPTP by inhibiting α-synuclein expression through the miR-21/Lamp2a axis (Su et al., 2016). In relation to Hsc70, two studies added miR-320 (Li et al., 2014) and miR-16-1(Zhang and Cheng, 2014) as direct regulators of Hsc70 expression, which negatively downregulated Hsc70 expression promoting α-synuclein aggregation in SH-SY5Y cells overexpressing α-synuclein, without affecting α-synuclein mRNA levels. Interestingly, miR-16 is a member of the miR-15/107 superfamily, a miRNA family highly dysregulated in Alzheimer’s disease (AD; Parsi et al., 2015). In this context, a preclinical study aimed to evaluate members of the superfamily miR-15/107 as potential drugs for AD, discovered that the brainstem of mice treated with a miR-16 mimic exhibited decreased α-synuclein protein levels (Parsi et al., 2015). This result was confirmed in HT22 cells, whereby overexpression of miR-16 downregulated α-synuclein protein levels (Parsi et al., 2015).

Another proteolytic pathway related with α-synuclein induced-toxicity is the autophagy-lysosomal pathway (ALP). The impact of ALP-associated miRNAs in α-synuclein expression was studied by Decressac et al. (2013) using rat midbrain overexpressing human wild-type α-synuclein. First, they demonstrated that α-synuclein toxicity is linked to impairment of the transcription factor EB (TFEB), a master regulator of the ALP controlled by mTOR signaling. In this context, AAV-mediated overexpression of miR-128 (which directly targeted TFEB) increased the formation of α-synuclein oligomers and the number of α-synuclein-positive axonal swellings, which resulted in α-synuclein-induced toxicity as revealed by a significant loss of nigral DA neurons, striatal innervation and DA levels, as well as development of motor deficits at 8 weeks after vector injection.

#### miR-133b

RhoA is a Rho family member that acts downstream of Rho-associated kinase (ROCK) and is a major regulator of the morphological events during apoptosis and neurite extension (Katoh et al., 1998; Shi and Wei, 2007). The fact that miR-133b was previously shown to promote neurite outgrowth and enhance neural function recovery after spinal cord injury and stroke by targeting RhoA (Liu et al., 2009; Yu et al., 2011; Xin et al., 2013), prompted Niu et al. (2016) to investigate the potential neuroprotective effect of miR-133b in the MPP^+^ model. In this scenario, Niu et al. (2016) reported that MPP^+^ treatment reduced miR-133b levels, increased RhoA expression and reduced neurite length in PC2 cells and rat DA neuron. Overexpression of miR-133b reversed the negative impact of MPP^+^ in neurite length and decreased RhoA protein level, although it had no impact on RhoA mRNA levels. Interestingly, ectopic expression of miR-133b in PC2 cells and primary neurons downregulated α-synuclein mRNA levels, both under baseline and MPP^+^ conditions. The authors attributed α-synuclein downregulation to miR-133 inhibition of RhoA, although this pathway has not been experimentally confirmed. Supporting this idea it has been previously reported that RhoA can directly modulate α-synuclein expression by activating the serum response element (SRF) transcription factor and GATA-2 transcription factor which regulates α-synuclein expression via occupancy at the intron-1 (Scherzer et al., 2008; Zhou et al., 2011).

#### miR-433

The discovery that SNPs located within the miR-433 binding sites in FGF20 gene were associated with PD (van der Walt et al., 2004; Haghnejad et al., 2015) triggered two studies to investigate the potential impact of miR-433 in α-synuclein expression (Wang et al., 2008; Schmitt et al., 2012). Wang et al. (2008) first demonstrated that miR-433 directly targets the FGF20 mRNA transcript and negatively regulates FGF20 protein translation. They showed that when SH-SY5Y cells were treated with the miR-433-target FGF20, α-synuclein protein levels were significantly increased compared to control cells. The authors suggest that FGF20 might regulate α-synuclein expression via FGF-receptor 1 (FGFR1), as it was previously demonstrated for FGF2 (Ohmachi et al., 2003; Rideout et al., 2003). Supporting this hypothesis, miR-433 did not bind to the α-synuclein 3′-UTR as reported with luciferase assays in Neuro2A and SK-N-SH cells (Schmitt et al., 2012).

### Synuclein-Induced Changes in miRNAs Expression

**Figure 5:**
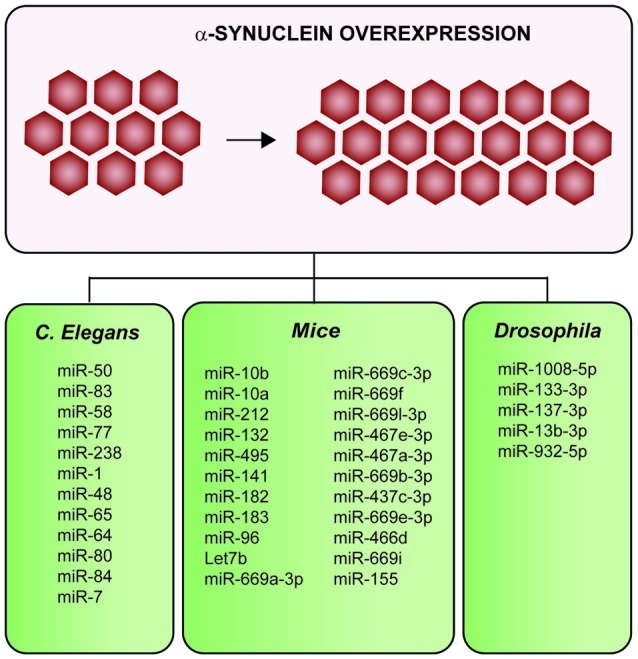
**Summary of the impacts on the miRNAs profile after α-synuclein-overexpression in multiple *in vivo* models.** Overexpression of α-synuclein induces alterations of several miRNAs in mice, *Drosophila* and *Caenorhabditis elegans* *(C. elegans)*.

In total, six studies report *in vivo* overexpressing α-synuclein models to investigate the impact of α-synuclein in miRNA expression (Figure 5; Table 2 and Supplementary Table 3; Gillardon et al., 2008; Asikainen et al., 2010; Ubhi et al., 2014; Kong et al., 2015; Schafferer et al., 2016; Thome et al., 2016). Four out of six studies used mice models (Gillardon et al., 2008; Ubhi et al., 2014; Schafferer et al., 2016; Thome et al., 2016), one was performed in *Caenorhabditis elegans* (Asikainen et al., 2010) and one in a *Drosophila model* (Kong et al., 2015). In all the species, overexpression of α-synuclein dysregulated several miRNAs (results summarized in Figure 5 and Supplementary Table 3).

One of the three mice model studies investigated alterations in the miRNA profile of early-symptomatic α-synuclein (A30P)-transgenic mice using microfluidic chip technology (LC Sciences, Houston, TX, USA; Gillardon et al., 2008). The study of 266 unique mature mouse miRNA probes using μParaflo microfluidic chip (#MRA-1002) revealed that five microRNAs were downregulated in early-symptomatic α-synuclein (A30P)-transgenic mice: mmu-miR-10b, mmu-miR-10a, mmu-miR-212, mmu-miR-132 and mmu-miR-495. Two out of three mice studies were focused on MSA mice models overexpressing α-synuclein in oligodendroglial cells (Ubhi et al., 2014; Schafferer et al., 2016). Of these two MSA models, one was a comparative study of frontal cortex from several neurodegenerative transgenic mice models: (i) two different models of MSA in which α-synuclein was expressed under the control of oligodendrocyte-specific MPG promoter (lines MBP1- hαsyn and MBP29-hαsyn, medium and high αsyn expression respectively); (ii) DLB/PD; (iii) AD; and (iv) tauopathy (Ubhi et al., 2014). The study revealed that 55 out of 88 microRNAs analyzed were dysregulated in both MSA models (MBP1 and MBP29) compared to non-transgenic animals, and five of these genes were disease specific (Supplementary Table 3). Surprisingly, the DLB/PD model expressing human α-synuclein under the control of the mThy1.2. promoter did not show significant differences. The second MSA study analyzed the striatum and SN in premotor MSA models overexpressing oligodendroglial α-synuclein in the third postnatal month using microarrays (Schafferer et al., 2016). The results showed that 33 miRNAs were dysregulated in the striatum and 59 miRNAs in the SN compared to control groups. Particularly, the miRNA family miR-437 was significantly enriched (*p* < 0.0001) among the up-regulated miRNAs (Figure 4 and Supplementary Table 3).

#### Neuroinflammation and miR-155

Considering the growing evidence that neuroinflammation plays a key role in the pathogenesis and progression of PD, Thome et al. (2016) investigated the impact of miR-155 expression, one of the key microRNA modulators of neuroinflammation, in the α-synuclein transgenic mice. Interestingly, adenovirus-mediated overexpression of α-synuclein (AAV2-Syn) enhanced the expression of miR-155 in the SN of α-synuclein-overexpressing mice compared to control (30% increment 2 weeks after transduction) and induced a 29.7 ± 6.6% loss of TH positive neurons in the SN 6 months after transduction. Reactive microgliosis markers Major Histocompatibility Complex Class II (MHCII) and CD68 were also increased in the AAV2-Syn transgenic mice. Interestingly, genetic deletion of miR-155 prevented the increments of MHCII and CD68 and markedly attenuated the TH positive neuronal loss in the SN of AAV2-syn transgenic mice. These results were confirmed *in vitro* using primary microglial murine cells. The authors first showed that microglial cells treated with fibrils of human wild-type α-synuclein exhibited increased levels of MHCII and inducible nitric oxide synthase (iNOS), while monomeric α-synuclein did not activate the inflammatory response. On the other hand, α-synuclein fibrils did not activate the inflammatory process in microglial cells derived from miR-155^−/−^ mice. However, miR-155 mimic treatment restored the inflammatory activity in miR-155^−/−^ microglial cells.

## Discussion

miRNAs both regulate and are regulated by α-synuclein expression, indicating the complex network between miRNAs and α-synuclein.

### miRNAs Regulate α-Synuclein Expression

Growing evidence indicates that increased levels of α-synuclein are toxic and may initiate a deleterious cascade of events leading to neuronal death in PD. However, the cause that triggers α-synuclein upregulation in PD is only understood in a small percentage of patients with duplications/triplications in the α-synuclein gene or SNPs in the α-synuclein promoter. miRNAs impact on α-synuclein expression raises the hypothesis that dysregulated miRNAs in PD patients are responsible for α-synuclein upregulation and/or accumulation. Supporting this idea, several studies have demonstrated that PD patients exhibited dysregulated miRNAs in brain (Kim et al., 2007; Cardo et al., 2013; Miñones-Moyano et al., 2013; Briggs et al., 2015; Hoss et al., 2016), blood (Margis et al., 2011; Martins et al., 2011; Khoo et al., 2012; Botta-Orfila et al., 2014; Burgos et al., 2014), cerebrospinal fluid (CSF; Burgos et al., 2014; Gui et al., 2015; Hossein-Nezhad et al., 2016) and medulla (Liao et al., 2013).

### Synuclein-Induced Changes in miRNAs Expression

α-synuclein overexpression impacts on miRNAs expression. Recent studies suggest that changes in miRNA expression can be directly linked to the pathophysiology of several diseases. Therefore, it is possible that the early α-synuclein overexpression linked to PD patients induces a stable miRNAs deregulation which can be the beginning of a process of neuronal death and the subsequent development of PD (Eacker et al., 2009). Supporting this idea, changes in miRNAs expression as a consequence of cellular damage and brain injury can be detected in the CSF and in the blood plasma/serum (Moldovan et al., 2014).

### miRNAs as Potential Therapeutic Opportunity in PD

Similar to other neurodegenerative diseases, there is still no treatment available that stops or halts the progression of PD; and symptomatic treatments are the only option for PD patients. In this context, a large proportion of therapeutic approaches under development are aimed to reduce α-synuclein expression levels.

Targeting miRNAs seems to be a potential therapeutic opportunity for PD. Indeed, multiple α-synuclein-targeting miRNAs (miR-7, miR-153, miR-214 and miR-133b) have displayed protective effects against the PD-like-induced toxins MPP^+^/MPTP. Strikingly, their effects are normally attributed to α-synuclein-independent mechanisms; for example, miR-7 may exert its protective effect by activating RelA, Nlpr3 and mTOR pathways. However, each miRNAs is unique and displays its own protective/deleterious effect: overexpression of miR-7 or miR-155 induced a protective effect in MPP^+^/MPTP models and α-synuclein-induced toxicity, while overexpression of miR-128 targeting TFEB exacerbated α-synuclein-induced toxicity in mice. Nonetheless, when considering miRNAs as therapeutic opportunities, one have to keep in mind that each miRNA can target various mRNA transcripts, rendering difficult to target a specific molecular way.

In addition to miRNAs that impact on α-synuclein expression, other miRNAs might play essential roles in the pathogenesis of PD. As an example, the list of miRNAs that target other PD-related genes such as LRKK2, Parkin and Pink becomes longer every year. Several reviews are available that illustrate the complex interplay of miRNAs in PD (Salta and De Strooper, 2012; Ma et al., 2013; Majidinia et al., 2016; Xie and Chen, 2016).

## Conclusion

This review article highlights that miRNAs regulate and are subject to regulation by α-synuclein. Considering the central role of α-synuclein in PD pathogenesis/progression, miRNAs are likely to play a crucial role at different stages of PD and might potentially be used in the future in new PD therapeutic approaches.

## Author Contributions

AR: conception and design of the work; acquisition, analysis and interpretation of data for the work; drafting, final approval of the version to be published and agreement to be accountable for all aspects of the work. CP: substantial contributions to the design of the work; drafting the work, revising it critically for important intellectual content; final approval of the version to be published and agreement to be accountable for all aspects of the work. CMS: substantial contributions to the conception of the work; revising it critically for important intellectual content; final approval of the version to be published and agreement to be accountable for all aspects of the work.

## Funding

This study was supported by the Sydney Medical School Foundation (University of Sydney).

## Conflict of Interest Statement

The authors declare that the research was conducted in the absence of any commercial or financial relationships that could be construed as a potential conflict of interest.
