# Supplementary material for: Role of microRNAs in the Regulation of α-Synuclein Expression: A Systematic Review
Source: Front Mol Neurosci. 2016 Nov 21;9:128. doi: 10.3389/fnmol.2016.00128 (PMC5116472; doi:10.3389/fnmol.2016.00128)
Supplement: Supplementary file 1 [file DataSheet_1.pdf]

Supplementary Material

Role of microRNAs in the Regulation of α-Synuclein Expression: a Systematic Review

Ariadna Recasens[1,2], Celine Perier[3], Carolyn M Sue[1,2*]

*** Correspondence:** Carolyn M Sue Carolyn.sue@sydney.edu.au

1 Supplementary methods.

Data extraction: all the included studies were divided into two groups: (i) overexpressing studies: studies using α-synuclein overexpressing models and (ii) standard studies. Relevant information from all included studies was extracted using two different extraction datasheets, depending on the category of the paper (overexpressing vs standard). The datasheet used for overexpressing studies extracted the following information: 1) Source: study ID and citation; 2) Objective; 3) Model used; 4) Methodology: RNA screening method, sample, RNA extraction method, studied miRNAs and quantification/statistic; 5) Results; 6) Conclusions and 7) Comments. The datasheet used for standards methods included: 1) Source: study ID and citation; 2) Subcategory: direct or indirect; 3) Objectives; 4) Model used: in vitro, in vivo and/or human samples and intervention; 5) Binding to α-synuclein 3'-UTR region: yes or no, methodology and results; 6) effect of miRNA in α-synuclein expression: yes or no, methodology and results; 7) effect of α-synuclein in miRNA: yes or no, methodology and results; 8) miRNA protective effect: yes or no, methodology and results; 9) Suggested pathway; 10) other results; 11) Summary of data; 12) Conclusions and 13) Comments.

Elegibility criteria: All the studies selected for the review satisfied the following PICOS selection criteria: (i) Population: miRNAs that impact directly or indirectly in α-synuclein expression; (ii) Interventions: miRNA mimics, miRNA inhibitors, miRNA overexpression and/or miRNA knockdown, as well as toxic treatments such as MPP+, MPTP, H2O2 and/or A53T α-synuclein overexpression to determine the miRNA protective effect; (iii) Comparator: proper control groups for each study were mandatory, such as untreated or empty vector treated groups when necessary; (iv) Outcomes: all quantitative studies, including western blot or immunohistochemistry for protein analysis, real-time PCR for mRNA expression, cell viability tests and luciferase reporter assays and (v) Study design: all original research publications describing the impact of miRNAs in α-synuclein expression were considered. No language or publication date restrictions were imposed

Study selection: Firstly, an over-inclusive screening by titles and abstracts was done to identify potential relevant studies. At this stage, irrelevant records, reviews, abstracts, editorials, letters, comments, perspective, reports, opinion and book chapter were removed. Full-text articles from the candidate studies were read and a second screening was done accordingly to the following exclusion criteria: (i) general single-nucleotide polymorphism (SNP) variants association with PD; (ii) general miRNA expression profile in PD patients; (iii) no study of α-synuclein expression; (iv) no study of miRNA expression and (v) no link between α-synuclein and miRNA.

2 Supplementary Tables

Supplementary table 1: Details of the review registration in The Joanna Briggs Institute website.

REGISTRATION DETAILS
**Centre**
The Joanna Briggs Institute (JBI)
**Primary Reviewer**
Ariadna Recasens, Kolling Institute, Royal North Shore Hospital, Clinical Medical School, University of Sydney
**Population**
microRNAs that regulates directly or indirectly the expression of the protein alpha-synuclein
**Intervention**
Intervention & Phenomena of Interest: The phenome of interest is how miRNA regulates alpha-synuclein expression. All animal models, in vitro models as well as studies using human sample will be considered in this systematic review.
**Context**
Comparator: The comparator or group control will depend on the study and will be stablished in each study. For instance, in a study where a wild-type animal is treated with a drug, the control group will be the wild-type without toxic treatment. In other instances, transgenic animals will be compared with wild-type animals.
**Outcome**
The outcomes will included quantitative results such as quantification of mRNA by RT-qPCR or protein level by Western blot, effect on cell viability (in vitro studies) or mortality (in vivo), nigrostriatal quantification.

Supplementary table 2: Summary of studies related with miRNAs that directly binds 3'-UTR α-synuclein and modulate α-synuclein expression. n/a= not applicable, FC: fold change;

	aSyn 3'-UTR binding	Effect of miR mimics			Effect of miR inhibitors			Protective effect
Ref	model	model	Protein reduction	mRNA reduction	model	Protein	mRNA	Model
miR-7								
(22)	HEK293T<br>SH-SY5Y	HEK293T	40-50%	34%	SH-SY5Y	1.5 FC	n/a	A53T aSyn NS20Y cells + H_2O_2
(23)	HEK293T<br>Primary neurons	HEK293T	30%	15%	n/a	n/a	n/a	n/a
(24)	HEK293	MIN6 cells	n/m	30%	MIN6 cells<br>Mir7a$^{2-/-}$ pancreatic islets	n/a<br>n/a	1.4 FC<br>1.5 FC	n/a
(25)	n/a	SH-SY5Y	n/m	n/a	n/a	n/a	n/a	SH-SY5Y + MPP$^+$
(26)	n/a	Not studied			Not studied			Murine neurons + MPP$^+$
(27)	n/a	A53T αSyn mice ANSC<br>A53T αSyn mice	80%<br>50%	n/a	n/a	n/a	n/a	A53T αSyn mice ANSC<br>A53T αSyn mice
(28)	n/a	n/a	n/a	n/a	n/a	n/a	n/a	MPTP and A53T aSyn mice
miR-153								
(23)	HEK293T<br>Primary neurons	HEK293T	19%	37%	n/a	n/a	n/a	n/a
(26)	Not studied	Not studied			Not studied			Murine neurons + MPP$^+$
(31)	HEK293TF	Not studied			Not studied			Not studied
miR-7/153								
(23)	HEK293T<br>Primary neurons	HEK293T<br>Primary neurons	46%<br>30-43% reduction	50%<br>n/a	n/a<br>n/a	n/a<br>n/a	n/a<br>n/a	n/a
miR-34b								
(32)	SH-SY5Y	SH-SY5Y	50%	60%	SH-SY5Y	2.2 FC	1.5 FC	n/a
miR-34c								
(32)	SH-SY5Y	SH-SY5Y	50%	60%	SH-SY5Y	1.7 FC	1.5 FC	n/a
miR-214								
(33)	SH-SY5Y	SH-SY5Y	n/a	80%	n/a	n/a	25 FC	MPP$^+$ / MPTP $^+$ resveratrol
miR-1643								
(31)	HEK293TF	n/a	n/a	n/a	n/a	n/a	n/a	n/a

Supplementary Table 3: Summary of miRNA profile studies using in vivo models overexpressing human α-synuclein

Model and sample	miRNA	Fold-change	Up or downregulated	Ref
**Mice model**				
**Early-symptomatic α-synuclein(A30P)-transgenic mice**<br>Sample: Brainstem	mmu-miR-10b<br>mmu-miR-10a<br>mmu-miR-212<br>mmu-miR-132<br>mmu-miR-495	-1.4<br>-0.9<br>-0.6<br>-0.5<br>-0.5	down<br>down<br>down<br>down<br>down	(49)
**Model: α-synuclein-transgenic mice mThy1.2. promotor**<br>Sample: Prefrontal cortex	No differences			(51)
**Model: Intermediate expresser MSA model – MBP1- hαsyn**<br>Sample: Prefrontal cortex	miR-141<br>miR-182<br>miR-183<br>miR-96<br>Let7b	24.0839<br>5.5117<br>4.8906<br>13.1546<br>2.2501	Up<br>Up<br>Up<br>Up<br>Down	(51)
**Model: High expresser MSA model – MBP29-hαsyn**<br>Sample: Prefrontal cortex	miR-141<br>miR-182<br>miR-183<br>miR-96<br>Let7b	12.9998<br>4.5015<br>3.1501<br>9.5715<br>2.3915	Up<br>Up<br>Up<br>Up<br>Down	(51)
**Model: MSA mice expressing α-synuclein under proteolipid protein promoter**<br>Sample**:** Striatum and SN	mmu-mir-669a-3<br>mmu-mir-669c-3p<br>mmu-mir-669f<br>mmu-mir-669l-3p<br>mmu-mir-467e-3p<br>mmu-mir-467a-3p<br>mmu-mir-669b-3p<br>mmu-mir-467c-3p<br>mmu-mir-669e-3p<br>mmu-mir-466d<br>mmu-mir-669i	1.7<br>1.7<br>1.8<br>1.6<br>1.7<br>1.5<br>1.7<br>1.6<br>1.4<br>1.4<br>1.4	Up<br>Up<br>Up<br>Up<br>Up<br>Up<br>Up<br>Up<br>Up<br>Up<br>Up	(53)
**Model: adenovirus-mediated overexpression of α-synuclein (AAV2-Syn)**<br>Sample: SN	mir-155	1.4	Up	(54)
C. Elegans				
**Model: α-synuclein(A53T)-transgenic *C. Elegan strain***<br>Sample: L4 stage	cel-miR-50<br>cel-miR-83<br>cel-miR-58<br>cel-miR-77<br>cel-miR-238<br>cel-miR-1<br>cel-miR-48<br>cel-miR-65<br>cel-miR-64<br>cel-miR-80<br>cel-miR-84<br>cel-miR-7	2.29<br>1.88<br>1.84<br>1.80<br>1.64<br>0.70<br>0.68<br>0.65<br>0.56<br>0.56<br>0.43<br>0.43	Up<br>Up<br>Up<br>Up<br>Up<br>Down<br>Down<br>Down<br>Down<br>Down<br>Down<br>Down	(50)
Drosophila				
**Model: Human α-synuclein(A30P)-transgenic *Drosophila***<br>Sample: heads	dme-miR-1008-5p<br>dme-miR-133-3p<br>dme-miR-137-3p<br>dme-miR-13b-3p<br>dme-miR-932-5p	1.22<br>1.30<br>1.25<br>1.49<br>1.28	Up<br>Up<br>Up<br>Up<br>Up	(52)
